# Effect of muscle fibre types and carnosine levels on the expression of carnosine-related genes in pig skeletal muscle

**DOI:** 10.1007/s00418-023-02193-6

**Published:** 2023-05-12

**Authors:** Claudia Kalbe, Katharina Metzger, Claude Gariépy, Marie-France Palin

**Affiliations:** 1 Research Institute for Farm Animal Biology, Institute of Muscle Biology and Growth, Dummerstorf, Germany; 2 Research Institute for Farm Animal Biology, Institute of Behavioural Physiology, Dummerstorf, Germany; 3 Agriculture and Agri-Food Canada, St-Hyacinthe Research and Development Centre, St-Hyacinthe, QC Canada; 4 Agriculture and Agri-Food Canada, Sherbrooke Research and Development Centre, Sherbrooke, QC Canada

**Keywords:** Carnosine-related genes, Gene expression, Immunohistochemistry, Muscle fibres, Myoblasts, Pig

## Abstract

It is generally accepted that carnosine (β-alanyl-l-histidine) content is higher in glycolytic than in oxidative muscle fibres, but the underlying mechanisms responsible for this difference remain to be elucidated. A first study to better understand potential mechanisms involved was undertaken (1) to determine whether differences in the expression of carnosine-related enzymes (*CARNS1*, *CNDP2*) and transporters (*SLC6A6*, *SLC15A3*, *SLC15A4*, *SLC36A1*) exist between oxidative and glycolytic myofibres and (2) to study the effect of carnosine on myoblast proliferative growth and on carnosine-related gene expression in cultured myoblasts isolated from glycolytic and oxidative muscles. Immunohistochemistry analyses were conducted to determine the cellular localization of carnosine-related proteins. Laser-capture microdissection and qPCR analyses were performed to measure the expression of carnosine-related genes in different myofibres isolated from the longissimus dorsi muscle of ten crossbred pigs. Myogenic cells originating from glycolytic and oxidative muscles were cultured to assess the effect of carnosine (0, 10, 25 and 50 mM) on their proliferative growth and on carnosine-related gene expression. The mRNA abundance of *CNDP2* and of the studied carnosine transporters was higher in oxidative than in glycolytic myofibres. Since carnosine synthase (*CARNS1*) mRNA abundance was not affected by either the fibre type or the addition of carnosine to myoblasts, its transcriptional regulation would not be the main process by which carnosine content differences are determined in oxidative and glycolytic muscles. The addition of carnosine to myoblasts leading to a dose-dependent increase in *SLC15A3* transcripts, however, suggests a role for this transporter in carnosine uptake and/or efflux to maintain cellular homeostasis.

**Supplementary Information:**

The online version contains supplementary material available at 10.1007/s00418-023-02193-6.

## Introduction

Carnosine is a histidine-containing dipeptide (β-alanyl-l-histidine) present in several mammalian organs and tissues, where it exerts multiple biochemical functions (Boldyrev et al. 2013). Carnosine is abundant in skeletal muscle (Mannion et al. 1992), and diet, gender, age, fibre type composition and the genetic background are major determinants that can modulate its muscle content (Harris et al. 2012; D’Astous-Pagé et al. 2017). In this tissue, carnosine acts as an antioxidant, a pH-buffering molecule (Baguet et al. 2010) and a regulator of calcium homeostasis (Dutka et al. 2012). Carnosine’s ability to increase skeletal muscle growth has also been suggested based on the observed increase in average daily gain values in pigs supplemented with l-carnosine (Bao et al. 2015) and on its capacity to increase the proliferation of porcine myogenic cells in vitro (Palin et al. 2020).

Carnosine is synthesized from β-alanine and l-histidine by the carnosine synthase (CARNS1) enzyme, and its degradation is performed by carnosine dipeptidases (serum carnosinase [CNDP1] and cytosolic non-specific dipeptidase [CNDP2]) (Boldyrev et al. 2013). Intracellular carnosine levels are also determined by β-alanine (proton-coupled amino acid transporter [PAT1/*SLC36A6*] and β-alanine/taurine transporter [TAUT/*SLC6A6*]) and carnosine/l-histidine (peptide/histidine transporters 1 [PHT1/*SLC15A4*] and 2 [PHT2/*SLC15A3*]) transmembrane transporters (Liu et al. 1992; Thwaites and Anderson 2011; Oppermann et al. 2019), all of which were previously detected in the porcine longissimus muscle (D’Astous-Pagé et al. 2017). Maintenance of carnosine homeostasis therefore depends on the gene expression and activity of the aforementioned enzymes and transporters, which can be modulated according to intracellular carnosine levels. A possible retro-control effect of carnosine on *CARNS1* messenger RNA (mRNA) levels has been suggested in the longissimus muscle of Duroc pigs having high muscle carnosine content (D’Astous-Pagé et al. 2017), and modulation of *CARNS1* mRNA expression in conditions of decreased or increased carnosine content in the mouse tibialis anterior muscle was reported by Everaert et al. (2013). However, evidence for a direct effect of carnosine on the expression of carnosine-related genes has never been demonstrated in muscle cells.

It is generally accepted that carnosine content increases with muscle glycolytic activity (Mora et al. 2008) and, when looking at individual muscle fibres, the ratio of type II (glycolytic) to type I (oxidative) carnosine content varies from 3.5 in the horse gluteus medius (Dunnett and Harris 1997) to 2.2 in the human vastus lateralis (Harris et al. 1998) and 2.1 in the camel gluteus medius (Dunnett and Harris 1997). Although carnosine content has never been investigated in pig individual muscle fibres, greater concentrations of carnosine were reported in skeletal muscles classified as predominantly glycolytic (e.g. longissimus dorsi and semimembranosus) than in oxidative muscles such as the masseter (Reig et al. 2013). On the other hand, the higher carnosine content found in the Duroc longissimus muscle, when compared with Landrace and Yorkshire pigs, could not be explained by differences in fibre type composition (D’Astous-Pagé et al. 2017).

The gene expression and activity of carnosine-related enzymes and transporters may account for some of the observed differences in carnosine content in glycolytic and oxidative muscle fibres, and variations in muscle fibre carnosine content may in turn affect these carnosine-related genes. However, no one has yet investigated whether glycolytic and oxidative muscle fibres present differences in the expression of carnosine synthase, carnosine dipeptidase and β-alanine, histidine and carnosine transporters. Moreover, it remains to be established whether the effect of carnosine on the expression of these genes differs according to the metabolic origin of the myogenic cells (e.g. glycolytic or oxidative muscles).

The present study was therefore undertaken (1) to determine whether there are differences in the expression (mRNA and protein) of carnosine-related genes in red (oxidative) and white (glycolytic) muscle fibres and (2) to study the effect of carnosine on proliferative growth and carnosine-related gene expression in primary myogenic cell cultures originating from predominantly glycolytic (longissimus dorsi [LD]) and oxidative (rhomboideus [RH]) muscles.

## Materials and methods

### Animals and tissue collection

A total of ten crossbred barrows (Duroc × Landrace-Yorkshire) were used for immunohistochemistry (IHC) and laser-capture microdissection (LCM) followed by quantitative polymerase chain reaction (qPCR) analyses. These pigs entered the Sherbrooke Research and Development Centre (SRDC, Agriculture and Agri-Food Canada, QC, Canada) at 20.5 ± 2.38 kg body weight (BW) and were sacrificed upon reaching 152.7 ± 11.90 kg BW. Pigs were fed ad libitum following a three-phase feeding program and had free access to water. Pigs were stunned with a captive bolt pistol prior to slaughter by exsanguination to ensure that the animals were killed humanely. The LD muscle was excised immediately after slaughter, and tissue samples were snap-frozen in liquid nitrogen and stored at −80 °C until IHC, LCM and gene expression analyses. Animal husbandry and slaughter procedures were conducted according to the recommended guidelines of the Canadian Council on Animal Care (2009) and approved by the local animal care committee of the SRDC.

For myogenic cell culture-related experiments (BrdU, DNA content and gene expression analyses), a total of five German Landrace female piglets from the experimental pig unit of the Research Institute for Farm Animal Biology (FBN, Dummerstorf, Germany) were used and slaughtered at 4–5 days of age. Four- to 5-day-old piglets were selected because they provide a better yield of satellite cells, in accordance with previous studies reporting a decline in the abundance of satellite cells with advancing age (Campion et al. 1981; Mesires and Doumit 2002). Piglets were sacrificed using exsanguination after stunning with a captive bolt pistol. The entire LD and RH muscles were rapidly collected and used for the isolation of satellite cells. The RH muscle is used as an oxidative (red) reference muscle with a high proportion of oxidative fibres (approximately 75%) and the LD is used as a glycolytic (white) reference muscle with a low proportion of oxidative fibres (approximately 35%) (Lösel et al. 2013). Experimental procedures were approved by the institutional Animal Protection Board at FBN.

### Laser-capture microdissection and qPCR analyses of carnosine-related genes in oxidative and glycolytic muscle fibres

**Fig. 1:**
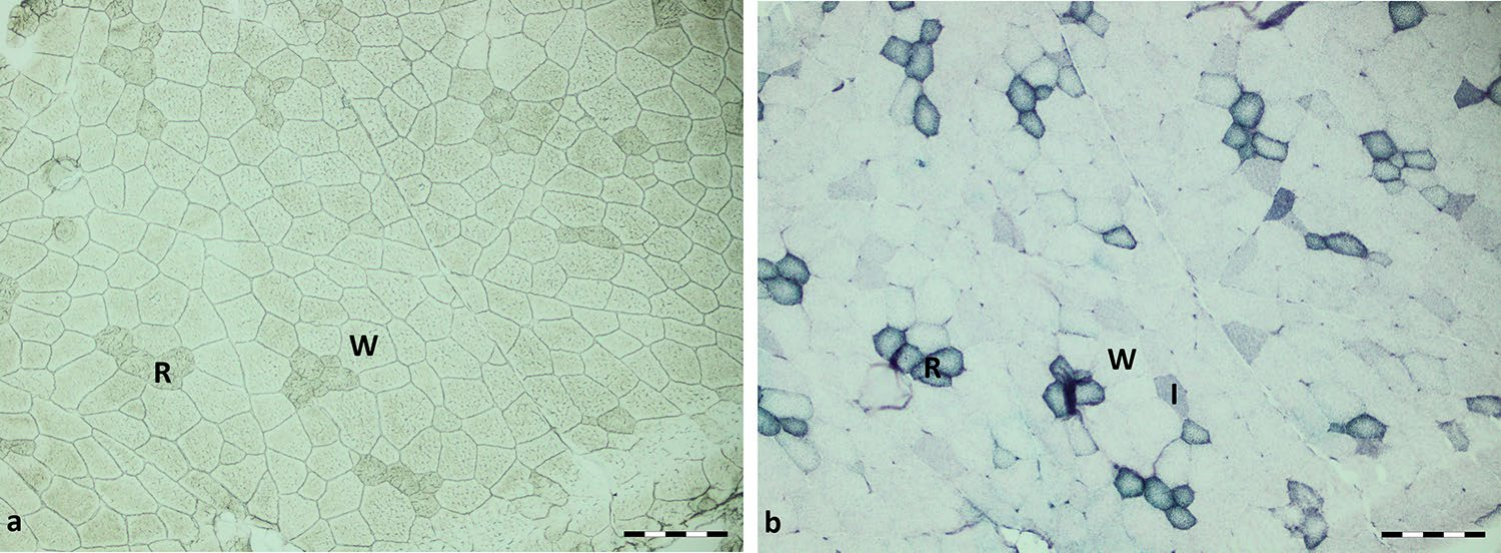
Representative sections of the longissimus dorsi (LD) muscle used for laser-capture microdissection (LCM). **a** Unstained section used to collect individual white glycolytic (W) and red oxidative (R) muscle fibres by LCM. **b** Adjacent section stained with NADH-tetrazolium reductase to allow the identification of different metabolic muscle fibre types (R, W and I [intermediate]), scale bar = 200 µm

The LCM (MicroBeam, PALM, Bernried, Germany) was performed as described by Albrecht et al. (2011) and Revskij et al. (2022), with some modifications. Briefly, frozen LD muscle tissue samples were cut into 10-µm-thick sections with a cryostat microtome (Leica CM 1950; Leica, Bensheim, Germany) at −20 °C. Membrane slides (MembraneSlide NF 1.0 PEN; Carl Zeiss, Göttingen, Germany) were activated with UV light for 60 min. Three serial sections were transferred to a membrane slide and air-dried before performing the dehydration procedure. The slide was then placed in 96% ethanol (−20 °C) and twice in absolute ethanol at room temperature (RT) for 30 s each. Thereafter, it was gently washed in xylene and placed in a second xylene container for 5 min before air-drying under a fume hood. The slide was immediately used for LCM. Red and white muscle fibres can be distinguished in microscopic cross section without specific staining, as demonstrated in Fig. 1, where two adjacent sections are shown, one without staining and the other one stained with NADH-tetrazolium reductase as described below. Some adjacent fibres of the same metabolic type (red or white) were marked, cut and collected in an adhesive cap (AdhesiveCap 500 opaque; Carl Zeiss). For each of the 10 animals, five slides were freshly prepared and processed, resulting in a total of 2500 red and 2500 white fibre cross sections, wherein the order of red and white fibre extraction was changed from slide to slide. The collected fibre cross sections per tube (500) were lysed in 100 µl RLT buffer (Qiagen, Hilden, Germany) containing 1 µl mercaptoethanol, shaken overhead and incubated for 30 min at RT before being stored at −80 °C. Total RNA isolation was performed with an RNeasy Micro Kit (Qiagen) according to the manufacturer’s instructions. For each animal, total RNA was isolated from white and red myofibres, resulting in five pooled tubes each. Total RNA was resuspended in 14 µl RNase free water. Complementary DNA (cDNA) synthesis was carried out with 7.5 µl of total RNA, a mixture (1:1) of random p(dN)_6_ and anchored-oligo (dT)_18_ primers (Roche, Mannheim, Germany) and 200 units of Moloney mouse leukaemia virus reverse transcriptase (M-MLV RT RNase H Minus Point Mutant, Promega, Mannheim, Germany) in 25 µl of the incubation buffer provided by the supplier, supplemented with 25 mM deoxy-NTPs (Roche) and 40 units of RNasin ribonuclease inhibitor (Promega). This mixture was incubated for 60 min at 42 °C. The cDNA was aliquoted and stored at −80 °C. For qPCR analyses, the diluted cDNA (1:4) was amplified in duplicate with the LightCycler FastStart DNA Master^PLUS^ SYBR Green I kit (Roche) in a 10 µl reaction volume. Primer information for the *CARNS1*, *CNDP2*, *SLC6A6*, *SLC15A3*, *SLC15A4* and *SLC36A1* genes is described in D’Astous-Pagé et al. (2017). Amplification and quantification of amplified products were performed in a LightCycler 2.0 instrument (Roche) under the following cycling conditions: pre-incubation at 95 °C for 10 min, followed by 40 cycles of denaturation at 95 °C for 15 s, annealing for 10 s at 60 °C, extension at 72 °C for 10 s and single-point fluorescence acquisition for 6 s to avoid quantification of primer artefacts. The melting peaks of all samples were routinely determined by melting curve analysis to ensure that only the expected products were generated. The quantification was performed with LightCycler software version 4.5 using the quantification module Absolute Quantification. To calculate the PCR efficiency, routine dilutions of gene-specific external standards (cloned PCR products) of known concentrations covering five orders of magnitude (5 × 10^–16^ to 5 × 10^–12^ g DNA) were co-amplified during each run.

### Detection and localization of carnosine-related proteins in oxidative and glycolytic muscle fibres

Histochemistry and IHC analyses were carried out to enable the identification of individual muscle fibre types and to detect the CARNS1, CNDP2, PHT2/SLC15A3 and PHT1/SLC15A4 proteins in red oxidative, intermediate and white glycolytic fibres. For these analyses, the LD muscle of crossbred barrows (Duroc × Landrace-Yorkshire; *n* = 10) described above were used. In addition, the semitendinosus (ST) muscles of two randomly selected 3-month-old domestic pigs (German Landrace females) were simultaneously stained as a reference for a mixed muscle with around 50% oxidative and 50% glycolytic fibres. Detailed information for housing, feeding management and muscle sample collection procedures for the 3-month-old domestic pigs are described in Lösel et al. (2013).

For the histochemistry analyses, serial transverse sections of both muscles (8 µm) were cut at −20 °C using a cryostat microtome (Leica CM 1950; Leica). One section was exposed to the reaction for NADH-tetrazolium reductase (Novikoff et al. 1961), which enables classification into metabolic fibre types (red oxidative, intermediate and white glycolytic) (Rehfeldt et al. 2008). Images were taken with an Olympus BX43 microscope (Olympus GmbH, Hamburg, Germany) equipped with an Olympus DP23 digital camera and using a 10× objective. Cell^D 5.1 software was used for image analysis (Olympus). Image acquisition details are as follows: image dimensions of 2080 (width) × 1544 (height) pixels and 24-bit depth, detector gain set at 1×, time and space resolution data 552.169 nm/pixel (*X*) and 552.169 nm/pixel (*Y*) with 96 pixels per inch. Exposure time for NADH-tetrazolium reductase was set at 33 ms.

For IHC analyses, the transverse frozen sections were mounted on poly-l-lysine-coated glass slides (Thermo Fisher Scientific, Darmstadt, Germany) and stored at −80 °C until staining. After thawing, sections were fixed in 4% formaldehyde (CNDP2, PHT2/SLC15A3) and/or in 0.3% H_2_O_2_ in methanol (CARNS1, PHT1/SLC15A4, CNDP2) and then washed with phosphate-buffered saline (PBS). Sections were pretreated with 10% bovine serum albumin (Sigma–Aldrich, Taufkirchen, Germany; PHT2/SLC15A3, PHT1/SLC15A4), 10% normal goat serum (Sigma–Aldrich; CARNS1) or Roti^®^ Block (Carl Roth, Karlsruhe, Germany; CNDP2) for 1 h to block unspecific binding of the secondary antibodies. The primary antibodies to PHT2/SLC15A3 (rabbit polyclonal to mouse SLC15A3, 1:50, ab113819, Abcam, Amsterdam, Netherlands), PHT1/SLC15A4 (chicken polyclonal to pig SLC15A4, 1:100, Immune Biosolutions, Sherbrooke, QC, Canada), CARNS1 (chicken polyclonal to pig CARNS1, 1:100, Immune Biosolutions) or CNDP2 (rabbit polyclonal to human CNDP2, 1:250, ab204351, Abcam) were added and sections were incubated in a humidified chamber overnight at 4 °C. More detail on primary and secondary antibodies is provided in Supplemental Table 1 (Supplemental Information file). Negative controls were incubated in PBS in place of primary antibody (Supplemental Fig. 1), and antibody specificity is presented in Supplemental Figs. 2, 3, 4 and 5. After washing with PBS, the sections were incubated with the secondary antibody Atto 488 goat anti-chicken immunoglobulin Y (IgY; VWR International, Mississauga, ON, Canada) for PHT1/SLC15A4 and CARNS1 staining at a dilution of 1:1000 for 1 h at RT. For PHT2/SLC15A3 and CNDP2 staining, the secondary antibody Alexa 488 goat anti-rabbit IgG (Abcam) at a dilution of 1:500 was used for 1 h at RT. Following washing in PBS (three times for 5 min each in the dark), slides were covered with Roti^®^ Fluor Care DAPI (Carl Roth) and a coverslip. Images were taken by the same experienced technician using a Leica DM 4000B microscope equipped with a DFC450 3.3 megapixel digital camera (Leica Microsystems, Wetzlar, Germany) and using a 10× objective. The band pass filter for excitation was set at BP450-490 nm (blue) and the long pass filter for emission was set at LP515 nm (green). QWin software (3.5.1) was used for image analysis (Leica Microsystems). Image acquisition was performed with the following parameters: image dimensions of 2088 (width) × 1550 (height) pixels and 24-bit depth, detector gain set at 48.86×, time and space resolution data 539.567 nm/pixel (*X*) and 539.567 nm/pixel (*Y*) with 96 pixels per inch. Exposure time was set at 1.0 s for CARNS1, 1.2 s for CNDP2, 986 ms for PHT2/SLC15A3 and 1.4 s for PHT1/SLC15A4. We did not conduct IHC analyses for the TAUT/SLC6A6 and PAT1/SLC36A1 proteins, as none of the commercially available antibodies raised against the human and rodent SLC6A6 and SLC36A1 proteins were suitable for detecting the porcine proteins. Western blot analyses were also performed to confirm antibody specificity in pig skeletal muscle and negative tissues. Detailed information on primary and secondary antibody characteristics (Supplemental Table S1) and western blot methods used are included in the Supplemental Information file. The CARNS1, SLC15A3 and SLC15A4 antibodies were used in a previous study, where we observed strong positive correlations between mRNA and protein abundance of CARNS1 (*r* = 0.98, *p* < 0.0001), SLC15A3 (*r* = 0.96,* p* < 0.0001) and SLC15A4 (*r* = 0.96, *p* < 0.0001) in pig skeletal muscle and where antibody specificity was confirmed in the longissimus muscle (D’Astous-Pagé et al. 2017).

**Table 1:** Effect of fibre type on the mRNA abundance (quantity units) of carnosine-related genes in the longissimus dorsi muscle

Gene^a^	Fibre type^b^				
	Red oxidative	White glycolytic	SEM^c^ red	SEM^c^ white	Fibre type *p* value
*CARNS1*	6.68	7.73	0.873	1.411	0.426
*CNDP2*	55.36	23.33	3.222	2.708	≤ 0.001
*SLC6A6*	3.66	0.75	0.853	0.026	0.007
*SLC15A3*	11.58	4.32	1.431	0.720	≤ 0.001
*SLC15A4*	3.19	1.26	0.311	0.225	≤ 0.001
*SLC36A1*	8.15	3.38	0.726	0.531	≤ 0.001

^a^*CARNS1* carnosine synthase 1, *CNDP2* carnosine dipeptidase 2, *SLC6A6* solute carrier family 6, member 6, *SLC15A3* solute carrier family 15, member 3, *SLC15A4* solute carrier family 15, member 4, SLC36A1 solute carrier family 36, member A1

^b^Data represent mean values of ten pigs with their corresponding SEM. Red and white fibres were isolated from the longissimus dorsi muscle using laser-capture microdissection

^c^Statistical analyses followed the usual verification of the normality of the residuals (Shapiro–Wilk tests), and the ANOVA was performed with heterogeneous variances. A non-parametric Friedman’s test was also used as confirmatory test

### Myogenic cell culture and carnosine treatments

Isolation and characterization of porcine satellite cells from the LD and RH muscles was performed as reported previously (Metzger et al. 2020). The isolated myogenic cells were aliquoted, stored in liquid nitrogen and used as passage 3 for the experiments. Previous studies have shown that satellite cells isolated from different muscle types possess preprogrammed characteristics of their muscle of origin (Ono et al. 2010; Zhang et al. 2022; Zhu et al. 2013). For each experiment, the myogenic cells were seeded in gelatin-coated plates at a density of 3000 cells per well for 96-well plates for proliferative responses (BrdU and DNA content analyses) or 150,000 cells in 6-mm cell culture dishes for RNA isolation (gene expression analyses). Cells were grown for 48 h in growth medium (GM: DMEM (Biochrom, Berlin Germany) containing 10% foetal bovine serum and 10% horse serum (Sigma–Aldrich), 1% glutamine (Carl Roth) and 1% penicillin/streptomycin (Biochrom)/amphotericin B (Sigma-Aldrich)) and treated with carnosine (0, 10, 25 and 50 mM; Sigma-Aldrich) for an additional 48 h. Selected carnosine concentrations and cell exposure time were chosen based on a previous study indicating similar cell proliferation curves up to 48 h for the 0, 10 and 50 mM carnosine doses (Palin et al. 2020). The 10 and 25 mM concentrations correspond to the physiological range for carnosine in pig skeletal muscle (Boldyrev et al. 2013), whereas the 50 mM carnosine represents a supra-physiological concentration. We recently demonstrated that these carnosine concentrations had no toxic effect in porcine myoblasts that were cultured up to 48 h (Palin et al. 2020).

### Myogenic cell proliferation assays and DNA content

The effect of carnosine and muscle type (LD vs RH) on the porcine myoblast proliferative response was measured by a BrdU colorimetric immunoassay (Cell Proliferation ELISA BrdU, colorimetric, Roche) allowing measurements of BrdU incorporation into newly synthesized DNA. After 48 h of cell growth in GM supplemented with carnosine (0, 10, 25 and 50 mM), myoblasts were incubated with 10 µM of the BrdU labelling solution for an additional 5 h. The culture medium was then removed by suction and cells were fixed using the provided FixDenat solution (Roche). Cells were incubated at RT for 2 h with the anti-BrdU-POD solution, allowing the formation of an immune complex, which was detected at an absorbance of 450 nm (reference wavelength 690 nm) after addition of the substrate solution. The stop solution option (H_2_SO_4_) was used before detection in order to stop the reaction. Absorbance was measured with a Synergy™ MX plate reader (BioTek, Bad Friedrichshall, Germany).

The DNA content was used as an equivalent for myogenic cell number and was measured as previously reported for piglet myoblasts (Mau et al. 2008a). For the BrdU assay and DNA content analyses, three independent experiments were conducted for each muscle (LD and RH), with *n* = 5 piglets used in each experiment (2 muscles × 3 experiments × 5 piglets) and carnosine concentrations in duplicate. The plate scheme was changed from experiment to experiment.

### qPCR analyses of carnosine-related genes in myogenic cells

The relative mRNA abundance of carnosine-related genes (*CARNS1*, *CNDP2*, *SLC6A6*, *SLC15A3*, *SLC15A4*, *SLC36A1*) was investigated in myoblasts after 48 h of cell growth with 0, 10, 25 and 50 mM carnosine. RNA isolation, cDNA synthesis and qPCR analyses were carried out and analysed as described previously (Kalbe et al. 2008, 2018) using the primers of D’Astous-Pagé et al. (2017). Data are expressed as arbitrary units after normalization with the endogenous reference gene *RPL32* (ribosomal protein L32, Wang et al. 2018). The *RPL32* mRNA expression was unaffected by carnosine, muscle type or the interaction of both (*p* > 0.10).

### Statistical analyses

Statistical analyses were performed with the MIXED procedure of SAS (2012; SAS Institute Inc., Cary, NC, USA). To analyse the effect of fibre type (red and white fibres) on the expression of carnosine-related genes (LCM + qPCR), an analysis of variance (ANOVA) was carried out. Statistical analyses followed the usual verification of the normality of the residuals (Shapiro–Wilk tests) to validate the ANOVA, and the analysis was also performed using a Friedman test as a confirmatory test.

For the BrdU, DNA content and qPCR analyses performed in myogenic cell cultures treated or not with carnosine, statistical analyses were undertaken using a 2 × 4 factorial analysis (2 muscles × 4 carnosine levels) randomized complete block design (3 experiments × 5 piglets = 15 blocks) that allowed comparisons between the LD and RH muscles (muscle effect) and the muscle × carnosine interaction. Partitioned analyses were performed for each muscle separately where the effect of carnosine was carried out, followed by Dunnett’s correction for comparing the 10, 25 and 50 mM carnosine treatments to the 0 mM control. Statistical significance was set at* p* ≤ 0.05 and tendencies at 0.05 < *p* ≤ 0.10.

## Results

### Expression of carnosine-related genes in oxidative and glycolytic muscle fibres

The qPCR analyses of carnosine-related genes in red oxidative and white glycolytic muscle fibres isolated from the LD muscle by LCM are presented in Table 1. A fibre type effect was observed for the *CNDP2*, *SLC6A6*, *SLC15A3*, *SLC15A4* and *SLC36A1* genes (*p* ≤ 0.01), with higher mRNA levels found in red oxidative than in the white glycolytic fibres. The red and white muscle fibres had similar *CARNS1* mRNA levels (*p* > 0.10).

### Immunohistochemical detection of carnosine-related proteins in porcine skeletal muscle fibres

**Fig. 2:**
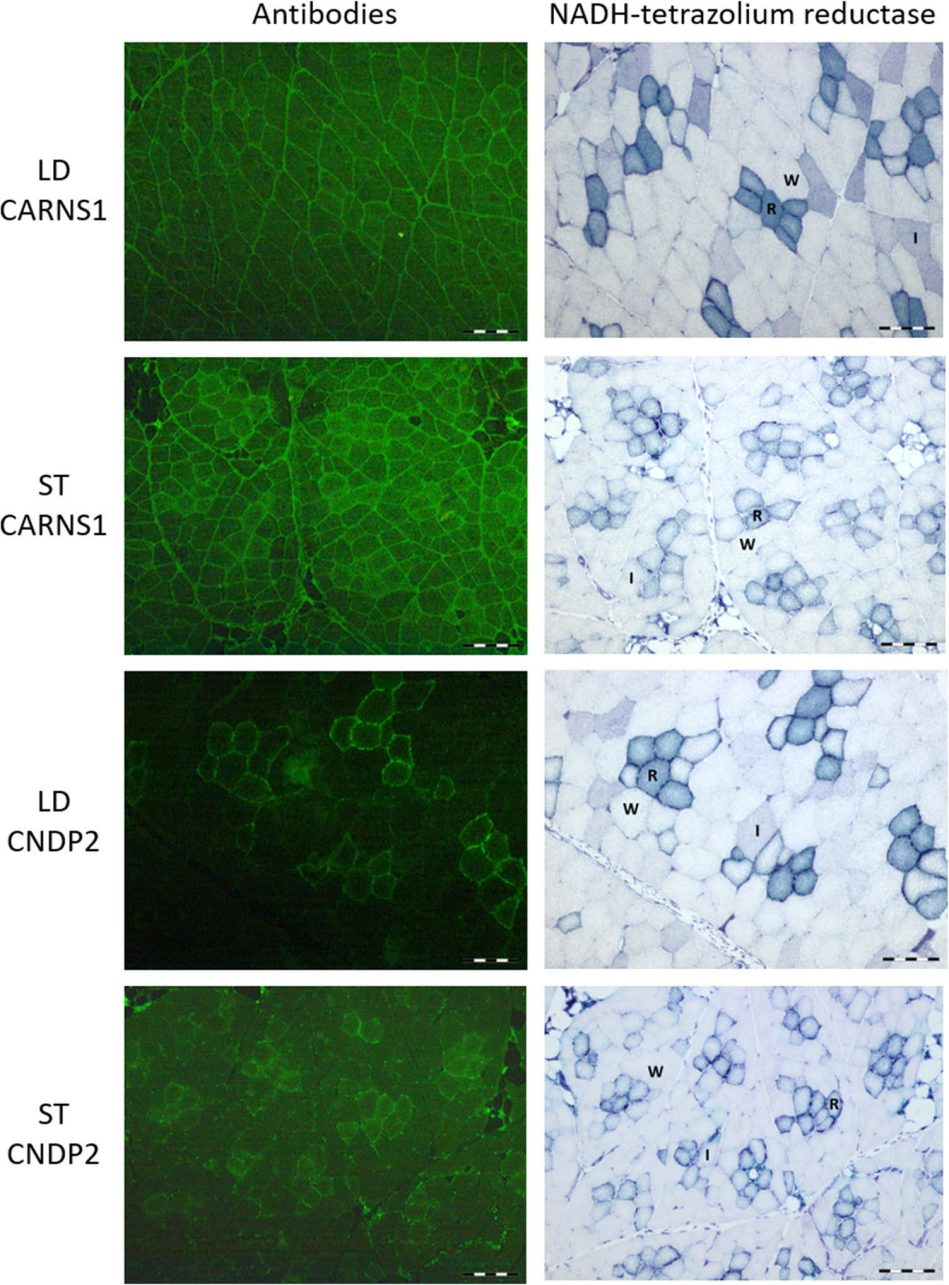
Immunohistochemical detection of carnosine synthase (CARNS1) and carnosine dipeptidase 2 (CNDP2) enzymes in serial cross sections of the longissimus dorsi (LD) and semitendinosus (ST) muscles. Serial sections were stained with anti-CARNS1 and anti-CNDP2 antibodies (left panels, green staining) or with a histochemical reaction based on NADH-tetrazolium reductase activity (right panels) to identify white glycolytic (W), intermediate (I) and red oxidative (R) muscle fibres. CARNS1 staining is mainly found at the periphery of W and R muscle fibres. A strong CNDP2 signal is also observed at the periphery of muscle fibres, with stronger intensity found in red oxidative fibres. Diffuse cytosolic CARNS1 and CNDP2 staining is also present in all fibre types, with slightly stronger intensity found in red oxidative fibres. Scale bar = 200 µm

**Fig. 3:**
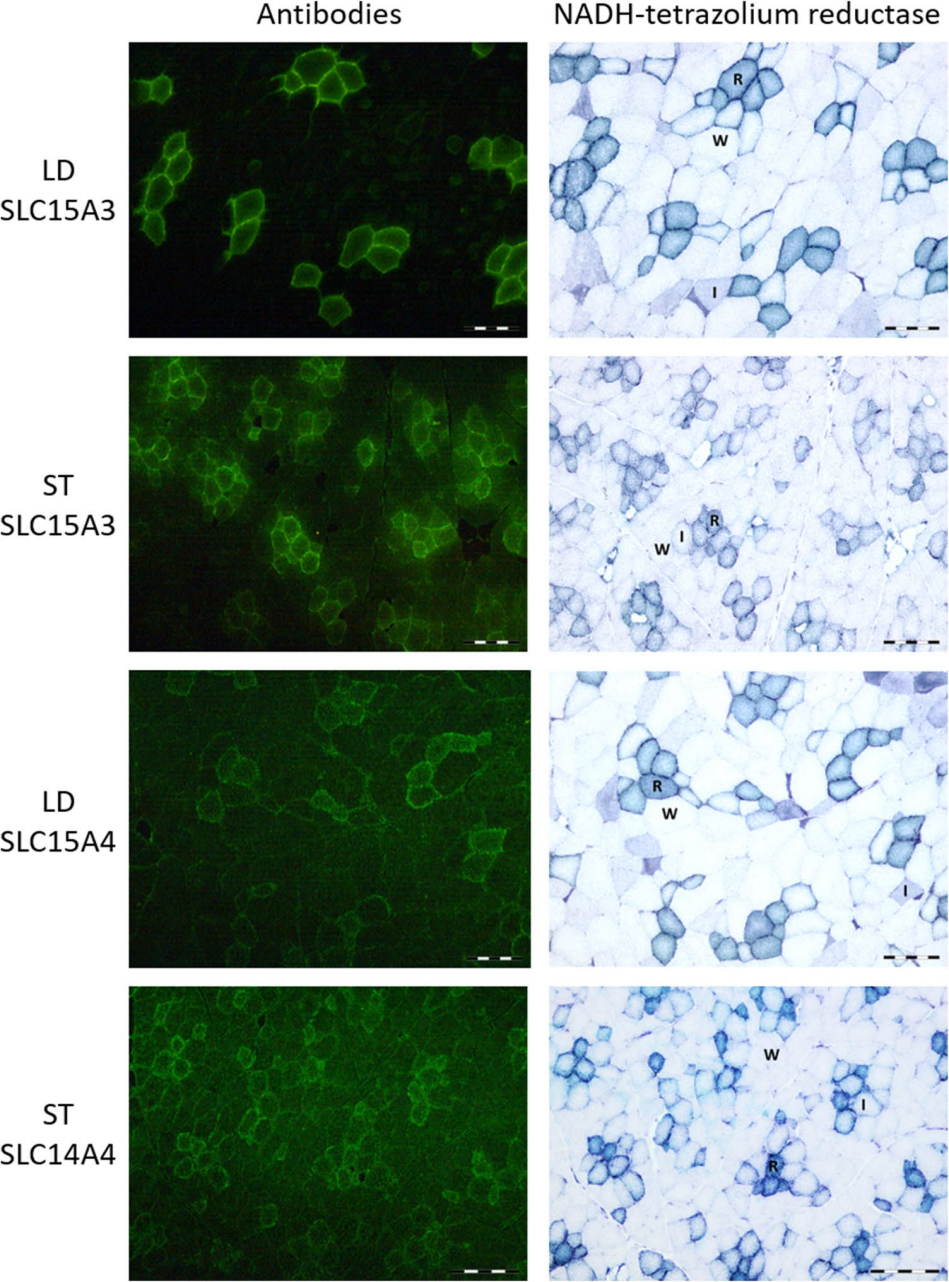
Immunohistochemical detection of PHT2/SLC15A3 and PHT1/SLC15A4 transporters in serial cross sections of the longissimus dorsi (LD) and semitendinosus (ST) muscles. Serial sections were stained with anti-SLC15A3 and anti-SLC15A4 antibodies (left panels, green staining) or with a histochemical reaction based on the NADH-tetrazolium reductase activity (right panels) to identify white glycolytic (W), intermediate (I) and red oxidative (R) muscle fibres. SLC15A3 and SLC15A4 signals are found at the periphery of muscle fibres, with a much stronger signal intensity present in red oxidative fibres for both muscles. A diffuse cytosolic staining is also present in all fibre types of the LD and RH muscles, with a stronger intensity found in red oxidative fibres. Scale bar = 200 µm

Detection and localization of the CARNS1, CNDP2, PHT2/SLC15A3 and PHT1/SLC15A4 proteins was performed by IHC in cross sections of the LD and ST muscles (Figs. 2 and 3). For the CARNS1 enzyme, a strong signal was detected at the periphery of white glycolytic (W) and red oxidative (R) fibres (Fig. 2). Diffuse cytosolic staining was also observed for CARNS1 in all fibre types (intermediate [I], R and W), with slightly stronger intensity found in red oxidative fibres. A strong CNDP2 signal was observed at the periphery of muscle fibres, with a stronger intensity found in R fibres. Diffuse and weaker CNDP2 cytosolic staining was found in all fibre types (Fig. 2). Again, slightly stronger cytosolic CNDP2 staining was observed in R fibres when compared to the I and W fibres of both muscles. For the PHT2/SLC15A3 and PHT1/SLC15A4 carnosine/histidine transporters, a strong signal was detected at the periphery of R fibres in LD and ST muscles (Fig. 3). For these two proteins, diffuse cytosolic staining was also present in all fibre types, with stronger intensity observed in R fibres (Fig. 3).

### Effect of carnosine on the proliferation of myoblasts isolated from the longissimus dorsi and rhomboideus muscles

**Fig. 4:**
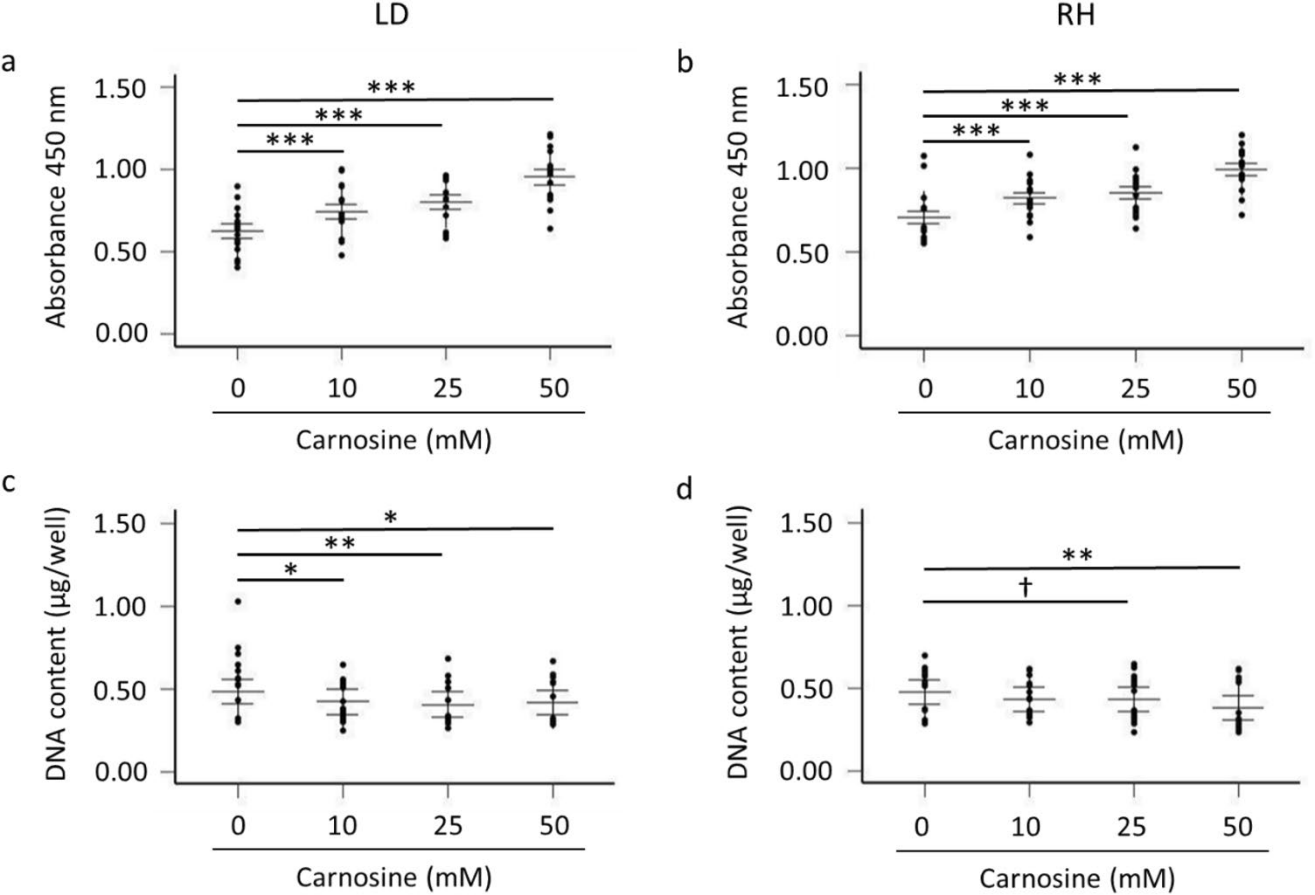
Effect of carnosine on myoblast proliferation (**a**, **b**) and cell number (**c**, **d**) after 48 h of growth. The effect of carnosine treatment on myoblast proliferation was assessed with the BrdU assay using myogenic cells isolated from the longissimus dorsi (LD; **a**) and rhomboideus (RH; **b**) muscles. The effect of carnosine on cellular DNA content (equivalent for myogenic cell number) was measured in myoblasts isolated from LD (**c**) and RH (**d**) muscles. Data represent mean values of *n* = 15 observations (5 pigs × 3 experiments) ± SEM. For both assays, the effect of carnosine was determined by comparing each dose of carnosine (10, 25 and 50 mM) to the 0 mM carnosine treatment on which a Dunnett’s correction was applied. BrdU SEM in LD = 0.046 and in RH = 0.036. DNA content SEM in LD = 0.074 and in RH = 0.073. ^†^0.05 < *p* ≤ 0.10; *p ≤ 0.05; **p ≤ 0.01; ***p ≤ 0.001

The effect of carnosine and muscle type on myoblast proliferation was evaluated by measuring the incorporation of BrdU into newly synthesized DNA after 48 h of carnosine treatment. This analysis revealed a significant overall carnosine effect on myoblast proliferation in the LD and RH muscles (*p* < 0.0001; Fig. 4a, b) and, in both muscles, the addition of 10 mM, 25 mM and 50 mM carnosine to the culture media significantly increased the myoblast proliferation when compared with the 0 mM carnosine treatment (*p* ≤ 0.0001). In addition, there was a muscle type effect, with higher absorbance values (450 mm) being observed in myoblasts isolated from RH (0.84) when compared with the LD (0.78) muscle (*p* < 0.001, SEM 0.032). There was no muscle × carnosine interaction.

The effect of carnosine on cell number (DNA content) was also investigated after 48 h of carnosine treatment (Fig. 4c, d). An overall carnosine effect was observed for myoblasts isolated from the LD and RH muscles (*p* < 0.05). For the LD muscle (Fig. 4c), the addition of 10 mM, 25 mM and 50 mM carnosine to the culture media decreased the DNA content values when compared with the 0 mM carnosine treatment (*p* < 0.05 for 10 mM and 50 mM carnosine; *p* < 0.01 for 25 mM carnosine). Lower DNA content values were also observed with the addition of 25 mM (tendency,* p* = 0.10) and 50 mM (*p* < 0.01) carnosine for myoblasts isolated from the RH muscle (Fig. 4d). Similar DNA content values were found between LD (0.433 µg/well) and RH (0.431 µg/well) muscles (muscle effect > 0.10, SEM 0.072) and there was no muscle × carnosine interaction.

### Effect of carnosine on carnosine-related gene mRNA abundance in cultured myoblasts isolated from the longissimus dorsi and rhomboideus muscles

Results for the mRNA abundance of carnosine-related genes in myoblast cell cultures from the LD and RH muscles are presented in Table 2. The qPCR analyses were performed after 48 h of cell growth in the presence of different doses of carnosine (0, 10, 25 and 50 mM). There was an overall effect of carnosine on the *SLC15A3* mRNA abundance in myoblasts isolated from LD and RH muscles (*p* < 0.001). For the LD muscle, higher *SLC15A3* mRNA levels were found in myoblasts that were exposed to 10 mM (*p* < 0.01), 25 mM (*p* < 0.0001) and 50 mM (*p* < 0.0001) carnosine when compared with the 0 mM treatment. For the RH muscle, increases in *SLC15A3* mRNA abundance were observed with the addition of 25 mM (*p* < 0.01) and 50 mM (*p* < 0.0001) carnosine. There was a muscle type effect on *SLC15A3* mRNA abundance, with higher abundance in LD than in the RH muscle (1.44 vs 1.30, *p* < 0.001, SEM = 0.150). There was a tendency for an overall carnosine effect on *CNDP2* mRNA abundance (*p* = 0.092) in the LD muscle. In addition, there was a muscle type effect (*p* < 0.01), with higher *CNDP2* mRNA abundance found in LD than in the RH muscle (1.12 vs 1.04, SEM = 0.037). A muscle type effect was also observed for the *SLC36A1* gene (*p* < 0.05), with higher mRNA abundance found in RH than in the LD muscle (1.17 vs 1.12, SEM = 0.077). There was no muscle × carnosine interaction for any of the studied genes.

**Table 2:** Effect of carnosine and muscle type on the relative mRNA abundance of carnosine-related genes in cultured myoblasts from the longissimus dorsi and rhomboideus muscles

Genes^a^	Muscle^b^	Carnosine (mM)^c^				SEM	Carn *p* value	Muscle* p* value	Carn × Muscle *p* value
		0	10	25	50				
*CARNS1*	LD	1.02	1.10	1.03	1.01	0.177	0.549	0.905	0.885
	RH	1.07	1.07	0.99	1.00	0.189	0.527		
*CNDP2*	LD	1.13	1.16	1.13	1.06	0.068	0.092	0.006	0.990
	RH	1.07	1.07	1.04	0.98	0.050	0.283		
*SLC6A6*	LD	1.08	1.02	0.98	1.01	0.120	0.296	0.426	0.829
	RH	1.11	1.09	1.01	0.99	0.107	0.113		
*SLC15A3*	LD	1.17	1.40**	1.49***	1.70***	0.173	< 0.001	< 0.001	0.844
	RH	1.08	1.21	1.35**	1.55***	0.129	< 0.001		
*SLC15A4*	LD	1.05	1.04	1.01	1.01	0.057	0.357	0.835	0.694
	RH	1.02	1.04	1.04	1.03	0.078	0.891		
*SLC36A1*	LD	1.08	1.10	1.12	1.15	0.083	0.175	0.041	0.895
	RH	1.16	1.18	1.18	1.18	0.097	0.945		

^a^*CARNS1 *carnosine synthase 1, *CNDP2* carnosine dipeptidase 2, *SLC6A6* solute carrier family 6, member 6; *SLC15A3* solute carrier family 15, member 3; *SLC15A4* solute carrier family 15, member 4; *SLC36A1* solute carrier family 36, member A1

^b^*LD* longissimus dorsi, *RH* rhomboideus

^c^Data represent mean values of *n* = 15 observations (5 pigs × 3 experiments). The mRNA abundance was normalized with the ribosomal protein L32 (*RPL32*) reference gene that was not affected by carnosine (*p* > 0.10) or the muscle type (*p* > 0.10). **p < 0.01; ***p < 0.0001

## Discussion

Several studies performed in different species have reported that muscle carnosine content is higher in white glycolytic than in red oxidative muscles (Baldi et al. 2021; Dunnett and Harris 1997; Mora et al. 2008). This finding is possibly linked with the higher pH buffering capacity of carnosine required in glycolytic fibres, which produce more lactic acid than oxidative fibres. The higher carnosine content generally observed in glycolytic fibres may also be associated with differences in the regulation of the abundance and/or activity of carnosine-related enzymes and transporters. To better understand the underlying mechanisms leading to the greater accumulation of carnosine in glycolytic fibres, we first investigated the mRNA abundance of carnosine-related genes in white glycolytic and red oxidative muscle fibres isolated with LCM. Study results showed similar mRNA abundance of *CARNS1* in red and white muscle fibres, a finding that was also confirmed at the protein level with IHC analyses. This may suggest that red and white fibres have similar capacity to synthesize carnosine from its precursors. This was indeed demonstrated by Hill et al. (2007), who reported a similar increase in carnosine content in type I (oxidative) and type II (glycolytic) muscle fibres after 10 weeks of β-alanine supplementation.

The greater abundance of the *CNDP2* transcript and its protein found in red oxidative fibres could lead to an increase in carnosine hydrolysis that would explain, at least in part, the lower carnosine content generally observed in these fibres. However, the importance of muscle carnosinase in controlling carnosine homeostasis has been questioned by indirect evidence of low carnosinase activity in skeletal muscle. This was indeed suggested by the slow washout profile (9 weeks) of muscle carnosine after cessation of β-alanine supplementation (Baguet et al. 2009) and by the optimal pH for CNDP2 activity that peaks at 9.5, whereas that of the skeletal muscle is at 7.1 (Lenney et al. 1985). Although these studies report important findings, further work assessing the CNDP2 activity in isolated muscle fibres is needed in order to obtain more direct evidence of increased carnosine hydrolysis in oxidative fibres (vs glycolytic fibres).

In the present study, the mRNA abundance of carnosine/l-histidine (*SLC15A3* and *SLC15A4*) and β-alanine (*SLC6A6* and S*LC36A1*) transporters was greater in red oxidative than in white glycolytic fibres, and this was also confirmed at the protein level when performing the IHC analyses. Since these transporters are involved in the cellular uptake of carnosine (Oppermann et al. 2019) and its constituents l-histidine (Yamashita et al. 1997) and β-alanine (Liu et al. 1992; Metzner et al. 2006), a higher mRNA abundance was rather expected in white glycolytic fibres, reported to accumulate more carnosine. A possible explanation may be that these transporters are also responsible for the release of carnosine, l-histidine and β-alanine from the cells (efflux) in order to maintain cellular homeostasis. This was suggested by Lindley et al. (2011), reporting on a potential efflux mechanism of PHT1/SLC15A4 for the histidine and Gly-Sar substrates in a transfected hPHT1-COS-7 cell model. Carnosine release was also demonstrated in primary cultures of glial cells (Bauer et al. 1982) and from the skeletal muscle of rats that had access to running wheels compared with sedentary rats (Nagai et al. 2003). Although it is tempting to hypothesize that red oxidative fibres present lower carnosine content because of greater carnosine and/or histidine cellular efflux, additional experiments are needed to determine whether such an active transporter-mediated process is involved.

Although the CARNS1, CNDP2, PHT2/SLC15A3 and PHT1/SLC15A4 proteins were previously detected in pig (D’Astous-Pagé et al. 2017), mice (Everaert et al. 2013) and human (Hoetker et al. 2018) skeletal muscle, their cellular localization has never been reported for this tissue. Moreover, very few studies have investigated their localization in other cell types. The CARNS1 enzyme has been observed in the cytosol and plasma membranes of neurons in the mice olfactory bulb (Wang-Eckhardt et al. 2020) and in intracellular, membranous and nuclear locations in human kidney, glial, neuronal and testis cells (www.proteinatlas.org). These results are in accordance with the observed localization of CARNS1 protein at the periphery of muscle fibres where myonuclei are located and in the cytosolic compartment of pig LD and ST muscles (current study). A similar cellular localization was found for CNDP2, with signals being observed in the cytosol and at the periphery of muscle fibres, with stronger staining intensities being observed in the red oxidative fibres of the LD and ST muscles. Cytosolic CNDP2 staining has been reported in human dopaminergic neurons (Licker et al. 2012) and in the cytoplasm and nucleus of *Drosophila melanogaster* S2 cells (Andreyeva et al. 2019), but the present study is the first to report staining at the periphery of muscle fibres. Accordingly, the Human Protein Atlas databank reported weak CNDP2 signals in the cytosolic, membranous and nuclear compartments of neuronal, kidney and testis cells (www.proteinatlas.org). The presence of CARNS1 and CNDP2 proteins in or in close proximity to the plasma membrane is quite surprising since both proteins lack transmembrane and secretion signal sequences and were predicted to be cytosolic enzymes (Drozak et al. 2010; Otani et al. 2005). The membrane-proximal staining in the muscle fibre cross section may suggest nucleus-associated expression due to the peripheral location of the nuclei in muscle fibres. Additional analyses using co-localization experiments with membrane-specific markers should help in determining the exact subcellular localization of these two proteins.

Carnosine is transported across cellular membranes through proton-coupled oligopeptide transporters (POTs) such as PEPT1/SLC15A1, PEPT2/SLC15A2, PHT2/SLC15A3 and PHT1/SLC15A4 (Kamal et al. 2009; Oppermann et al. 2019) and, in addition to carnosine, the PHT2/SLC15A3 and PHT1/SLC15A4 transporters are also involved in the cellular uptake of histidine (Bhardwaj et al. 2006; Sakata et al. 2001). The PEPT1/SLC15A1 and PEPT2/SLC15A2 transporters were not included in the present study because of undetectable transcripts (SLC15A1) or very low transcript levels (SCL15A2) in mouse, human and pig skeletal muscles (Everaert et al. 2013; D’Astous-Pagé et al. 2017). Intracellular lysosomal localization has been reported for PHT1/SLC15A4 (Sasawatari et al. 2011) and PHT2/SLC15A3 (Sakata et al. 2001) proteins in a number of cell lines (HEK-293 T, BHK, COS-7 and RAW264.7). However, ectopic overexpression of PHT1/SLC15A4 and PHT2/SLC15A3 was conducted for all of these cell lines, which may not reflect the cellular localization found in vivo. In fact, the human PHT1/SLC15A4 protein was found in the plasma membrane of villous epithelial cells from the small intestine (Bhardwaj et al. 2006) and in the nuclei of nasal epithelial cells (Agu et al. 2011) and, to our knowledge, no one has yet reported the presence of PHT1/SLC15A4 and PHT2/SLC15A3 in the lysosomal compartment of human, mouse or pig skeletal muscle tissues. The only report of PHT1/SLC15A4 and PHT2/SLC15A3 protein localization in skeletal muscle comes from the Human Protein Atlas databank (www.proteinatlas.org), with cytoplasmic and membranous localization being observed in myocytes. In the present study, the strong PHT2/SLC15A3 and PHT1/SLC15A4 signals observed at the periphery of red oxidative muscle fibres suggest implications for these proteins in the trans-sarcolemma transport of carnosine and histidine. The presence of a weak and more diffuse signal for PHT2/SLC15A3 and PHT1/SLC15A4 in the cytosol of pig muscle fibres may also suggest intracellular membrane-bound localization. As previously reported for the rat PHT1/SLC15A4 and PHT2/SLC15A3 proteins (Sakata et al. 2001), the presence of a di-leucine-based motif at the N-terminus of the porcine PHT2/SLC15A3 (GERRP**LL**A) and PHT1/SLC15A4 (GERAP**LL**G) proteins may allow protein sorting to the endosomes and lysosomes. However, additional IHC experiments with lysosomal-specific markers are needed to elucidate the exact subcellular localization of the porcine PHT2/SLC15A3 and PHT1/SLC15A4 proteins in the cytosolic compartment of muscle fibres.

The addition of 10, 25 and 50 mM carnosine to cultured myoblasts led to a decline in cell number, as indicated by lower DNA content values (vs 0 mM carnosine) in the LD and RH muscles. A similar observation has been reported when using the 3-(4,5-dimethyl-thiazol-2-yl)-5-(3-carboxymethoxyphenyl)-2-(4-sulfophenyl)-2H-tetrazolium (MTS) assay as a surrogate for porcine myoblast viability and, in both studies, this was accompanied by an increase in cell proliferation (at 10, 25 and 50 mM carnosine in the current study and at 10 and 25 mM carnosine in Palin et al. 2020). Seidel et al. (2022) recently reported that the effect of carnosine on cell viability differs between malignant and non-malignant cells, with a minor effect of carnosine in human fibroblasts and significant reduction of cell viability when carnosine is added to glioblastoma cell cultures. The anti-neoplastic effect of carnosine was also confirmed in gastric and colon cancer cells (Shen et al. 2014; Iovine et al. 2012). The similar reduction in cell viability found in cancer cells and satellite-derived myoblast cells (current study) when exposed to carnosine may be explained, at least in part, by common features shared by these two cell types. Indeed, satellite cells are considered as adult muscle stem cells that, just like cancer cells, have the capacity to proliferate and the potential to differentiate (Yin et al. 2013). The dose-dependent stimulation of myoblast proliferation with the addition of carnosine, while observing a concomitant decrease in cell viability/DNA content, may be caused by DNA repair mechanisms. Such compensatory DNA synthesis has already been described after the addition of high concentrations of the phytoestrogens genistein (from 10 µM) and daidzein (100 µM) in porcine myoblasts (Mau et al. 2008b). Alternatively, proliferative growth of the remaining healthy myoblasts in the carnosine-supplemented cell culture may be an explanation. The addition of carnosine may also increase the cellular lifespan as previously observed in human senescent myoblasts showing increased replicative potential after carnosine supplementation to the culture media (Maier et al 2012).

The gene expression analyses of carnosine-related genes in cultured myoblasts treated with different concentrations of carnosine revealed a clear dose-dependent increase in *SLC15A3* mRNA abundance in both muscles (LD and RH), whereas there was no carnosine effect on the *SLC15A4* gene. The lack of effect of carnosine on *SLC15A4* mRNA abundance was unexpected, since small interfering RNA (SiRNA)-mediated knockdown of both receptors (*SLC15A3* and *SLC15A4*) was found to significantly reduce carnosine transport in human glioblastoma cells (Oppermann et al. 2019). On the other hand, oral supplementation of carnosine to mice for 8 weeks reduced *SLC15A3* mRNA expression levels by 34% in the tibialis anterior muscle and had no effect on *SLC15A4* mRNA abundance (Everaert et al. 2013). Discrepancies among studies may be explained by species (human, mice and pig) and/or tissue (glioblastoma, skeletal muscle) specific effects. Exposure time to carnosine may also affect the transporter transcriptional response, with a possible retro-control effect of carnosine on *SLC15A3* mRNA abundance after 8 weeks of carnosine supplementation (Everaert et al. 2013) and an upregulation of *SLC15A3* transcript abundance in short-term experiments (48 h in current study) where carnosine levels would remain within the limits of cellular homeostasis. The above studies also suggest that SLC15A3 would be the main receptor involved in the trans-sarcolemma transport of carnosine in muscle cells, since SLC15A4 was unaffected by either long-term (Everaert et al. 2013) or short-term (current study) exposure to carnosine.

A possible retro-control effect of carnosine on its own synthesis has been suggested in previous studies. This was first proposed by Everaert et al. (2013), who reported lower *CARNS1* mRNA abundance in conditions of high muscle carnosine content and, on the contrary, higher *CARNS1* gene expression with reduced carnosine content. A similar observation was made in the pig skeletal muscle, with lower *CARNS1* mRNA abundance found in pigs having high muscle carnosine content (D’Astous-Pagé et al. 2017). However, this was only observed in the Duroc breed, which had the highest concentrations of muscle carnosine, and not in Landrace or Yorkshire pigs, which presented lower carnosine values. This has led to the hypothesis that a certain threshold of muscle carnosine must be reached before triggering a carnosine-inhibitory effect on *CARNS1* transcription. Current results indicate that *CARNS1* mRNA abundance is not affected by the addition of carnosine to cultured myoblasts isolated from LD or RH muscles. A similar observation was made in mice skeletal muscle, where oral supplementation of carnosine for 8 weeks increased muscle carnosine content by 48%, but had no effect on *CARNS1* mRNA abundance (Everaert et al. 2013). However, an increase in *CARNS1* mRNA abundance was observed (tendency) in the skeletal muscle when these mice were instead supplemented with β-alanine over the same period of time (Everaert et al. 2013). An increase in *CARNS1* mRNA abundance was also reported in skeletal muscle of broilers that were supplemented with β-alanine for 42 days (Qi et al. 2018). Together, these results suggest that carnosine synthase substrates such as β-alanine, but not its product carnosine, can modulate the abundance of *CARNS1* transcript levels. Results from Barca et al. (2019) further suggest that the regulation of *CARNS1* transcript abundance may vary according to the tissue analysed, since an increase in *CARNS1* mRNA abundance was observed in the brain of mice supplemented with carnosine. Lastly, the absence of carnosine effect on *CARNS1* mRNA abundance in cultured myoblasts could be because carnosine doses used in the current study were not high enough to cause a retro-control effect.

The *CNDP2* transcript is present in human, mouse and pig skeletal muscle, whereas that of the serum carnosinase *CNDP1* is undetectable or barely detectable (D’Astous-Pagé et al. 2017). Although an overall effect of carnosine (tendency) is observed on *CNDP2* mRNA abundance in cultured myoblasts isolated from the LD muscle only, the comparison of each carnosine dose to the control (0 mM carnosine) did not reach statistical significance. Very few studies have investigated the effect of carnosine on *CNDP2* transcript abundance. In accordance with our findings, there was no significant change in *CNDP2* mRNA abundance in the mouse brain after 2 weeks of carnosine supplementation (Barca et al. 2019). Intriguingly, mice supplemented with β-alanine for 8 weeks showed a 27% increase in *CNDP2* mRNA levels in the skeletal muscle, while supplementation with carnosine resulted in a 39% decrease (Everaert et al. 2013). Although discrepancies among the abovementioned studies may be explained by species (mice vs pigs), tissue (skeletal muscle vs brain) and/or time (48 h, 2 weeks and 8 weeks) effects, the importance of CNDP2 in controlling intramuscular carnosine homeostasis remains to be demonstrated. According to Everaert et al. (2013), the ability of CNDP2 to hydrolyse carnosine in skeletal muscle would be quite limited due to its optimal activity being reached at pH 9.5 and the slow carnosine washout in skeletal muscle upon cessation of β-alanine supplementation (Baguet et al. 2009; Lenney et al. 1985).

The lack of effect of carnosine on *SLC6A6* and *SLC36A1* mRNA abundance in cultured myoblasts from both muscles (LD and RH) was expected, as these two transporters are rather involved in β-alanine and histidine cellular uptake (Yamashita et al. 1997; Liu et al. 1992; Metzner et al. 2006). Oral supplementation of β-alanine for 8 weeks significantly increased the expression of *SLC6A6* in mice skeletal muscle and had no effect on *SLC36A1*, thus suggesting a dominant role for SLC6A6 in the cellular uptake of β-alanine (Everaert et al. 2013). Surprisingly, a 21% increase in *SLC6A6* mRNA abundance (tendency) was also observed in the skeletal muscle of mice supplemented with carnosine (Everaert et al. 2013). Since PAT1/SLC6A6 has never been shown to be involved in carnosine transport, it can be hypothesized that the observed increase in its transcript abundance is related to an increase in cellular efflux of β-alanine and/or histidine in order to maintain carnosine homeostasis during long-term supplementation. We were unable to observe such an increase in *SLC6A6* mRNA abundance in cultured myoblasts, possibly due to the short-term exposure to carnosine (48 h) or its limited effect on carnosine cellular homeostasis in pig skeletal muscle.

## Conclusions

The present study identifies for the first time the cellular localization of carnosine-related proteins in white glycolytic and red oxidative muscle fibres and presents a clear fibre type effect with higher mRNA abundance of *CNDP2*, *SLC6A6*, *SLC15A3*, *SLC15A4* and *SLC36A1* in red compared with white myofibres. Carnosine synthase mRNA abundance was not affected by either fibre type or the addition of carnosine to myoblast culture, which serve as an in vitro model for metabolic fibre types, thus suggesting that its transcriptional regulation would not be the main process by which carnosine content differences are determined in oxidative and glycolytic muscles. The dose-dependent effect of carnosine on the mRNA abundance of *SLC15A3* in myoblasts and its higher expression in oxidative fibres suggest a role for this transporter in carnosine uptake and/or efflux in order to maintain cellular homeostasis. The unexpected increase in *SLC6A6*, *SLC15A3*, *SLC15A4* and *SLC36A1* transcripts (LCM) and proteins (IHC) in red oxidative fibres that present lower carnosine content further suggests that cellular efflux mechanisms should be considered as important points of regulation in controlling carnosine homeostasis. It is also important to mention that other alternative mechanisms, such as the enzymes involved in β-alanine synthesis (GADL1) or degradation (ABAT and AGXT2), may also affect carnosine homeostasis in the skeletal muscle (Mahootchi et al. 2020; Blancquaert et al. 2016), although this has not yet been demonstrated in pigs. Although the present study has allowed us to better understand fibre type-specific carnosine-related gene expression and protein localization, additional information is needed on the mechanisms controlling the synthesis and degradation, as well as the cellular uptake and release of carnosine, histidine and β-alanine in white and red muscle fibres.

## Supplementary Information

Below is the link to the electronic supplementary material.

Supplementary file1 (PDF 763 KB)

## Data Availability

All data supporting the findings of this study are included in the published article. Additional information is available from the corresponding author upon reasonable request.
