# Supplementary material for: Effect of muscle fibre types and carnosine levels on the expression of carnosine-related genes in pig skeletal muscle
Source: Histochem Cell Biol. 2023 May 12;160(1):63–77. doi: 10.1007/s00418-023-02193-6 (PMC10313551; doi:10.1007/s00418-023-02193-6)
Supplement: Supplementary file 1 — Supplementary file1 (PDF 763 KB) [file 418_2023_2193_MOESM1_ESM.pdf]

Supplemental Information

Effect of muscle fibre types and carnosine levels on the expression of carnosine-related genes in pig skeletal muscle

Claudia Kalbe[1] · Katharina Metzger[2] · Claude Gariépy[3] · Marie-France Palin[4]*

[1]Research Institute for Farm Animal Biology, Institute of Muscle Biology and Growth, Dummerstorf, Germany

[2]Research Institute for Farm Animal Biology, Institute of Behavioural Physiology, Dummerstorf, Germany

[3]Agriculture and Agri-Food Canada, St-Hyacinthe Research and Development Centre, St-Hyacinthe, Québec, Canada

[4]Agriculture and Agri-Food Canada, Sherbrooke Research and Development Centre, Sherbrooke, Québec, Canada

*** Corresponding author:** Marie-France Palin, Mariefrance.palin@agr.gc.ca

1- Immunohistochemistry analyses were conducted to show the absence of non-specific binding when serial sections are incubated in PBS, in place of primary antibodies (negative controls).

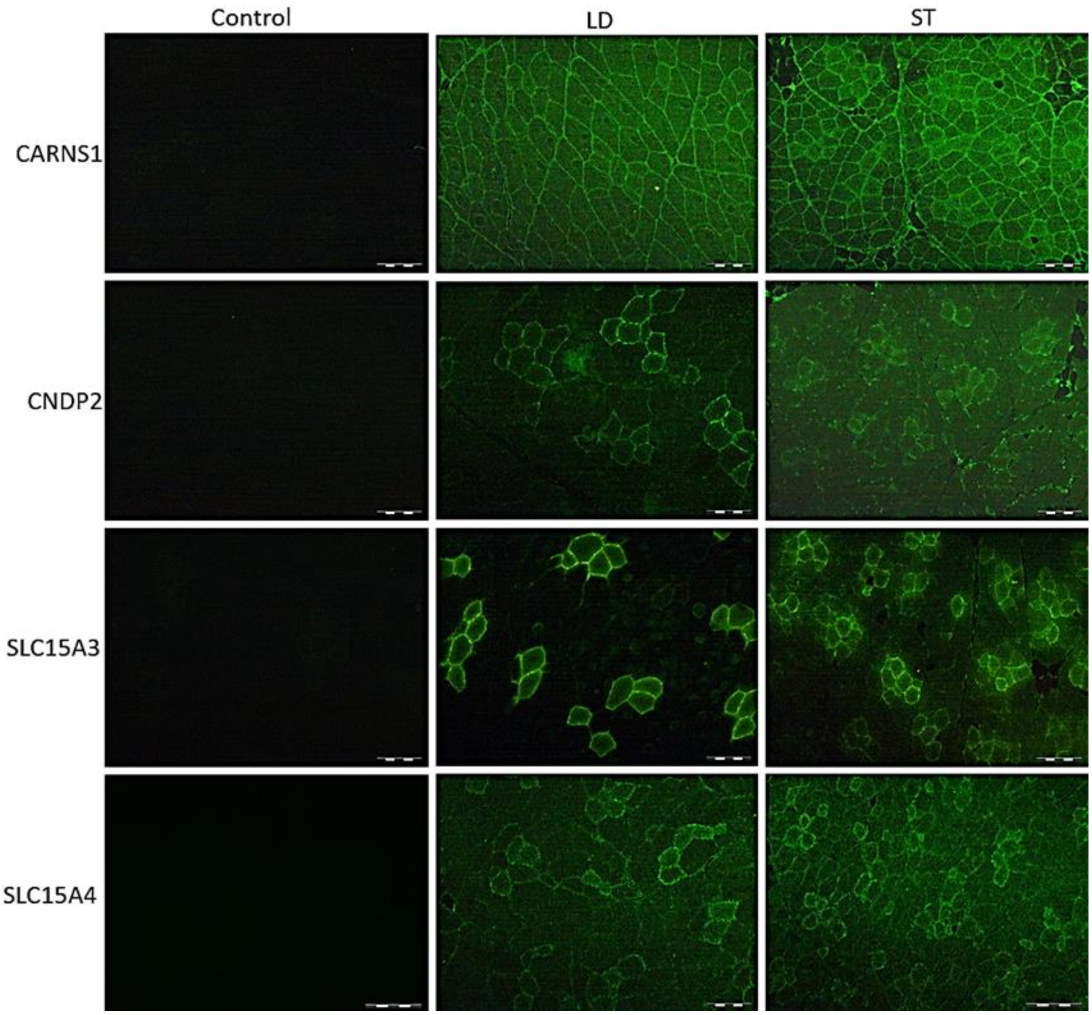

Supplementary Fig. 1 Immunohistochemical detection of carnosine synthase 1 (CARNS1), carnosine dipeptidase 2 (CNDP2) and PHT2/SLC15A3 and PHT1/SLC15A4 transporters in serial cross sections of the *longissimus dorsi* (LD) and *semitendinosus* (ST) muscles. Left panels: non-specific binding of secondary antibodies (negative controls), serial sections were incubated in PBS in place of primary antibodies. Serial sections of the LD (center) and ST (right panels) muscles were stained with primary and secondary antibodies (green staining). scale bar = 200 µm

2- Primary antibodies specificity

Supplemental Materials and methods

Western blot analyses were conducted to validate primary antibodies specificity towards the porcine CARNS1, CNDP2, SLC15A3 and SLC15A4 proteins. Total protein extraction was performed with the T-PER™ tissue protein extraction reagent or the Mem-PER™ Plus membrane protein extraction kit (Thermo Scientific, Burlington, ON, Canada) for CARNS1, CNDP2 and SCL15A4 in the presence of protease and phosphatase inhibitors (Phenylmethylsulfonyl fluoride (PMSF, 1X) to inhibit serine and cysteine proteases (Sigma-Aldrich, St. Louis, MO); Halt™ Protease inhibitor cocktail (1X) to inhibit serine, cysteine and aspartic acid proteases and aminopeptidases (Thermo Scientific); Halt™ inhibitor cocktail (1X) to inhibit serine/threonine and tyrosine phosphatases (1X) (Thermo Scientific)). Protein extraction for SLC15A3 was carried out as previously reported (Metzger et al., 2021). For serum and plasma samples, an additional step was carried out to remove albumin and IgG using the PierceTM Albumin/IgG Removal kit (Thermo Scientific) for CARNS1, CNDP2 and SLC15A4 or the acetonitrile-methanol-water (AMW) protocol for SLC15A3 (Tomascova et al., 2019). Albumin and IgG can represent up to 70% of total serum and plasma proteins and often mask lower quantity proteins. Protein content was determined using the Bio-Rad DC protein assay (BIO-RAD, Mississauga, ON, Canada) for CARNS1, CNDP2 and SLC15A4, or as previously described for SLC15A3 (Metzger et al., 2021).

Extracted proteins were loaded (20 or 30 µg per well) and separated by electrophoresis (1 h 30 at 125 Volts) using 10% tris-glycine mini gels (Novex™ WedWell™ 10%, mini protein gels, Thermo Scientific) under reducing conditions. The Mini Gel Tank electrophoresis chamber system (Thermo Scientific) was used with the following running buffer: 25 mM tris, 192 mM glycine and 0.1% SDS (1 h at 125 Volts). The Mini Gel Tank chamber system was then used for wet transfer of proteins on PVDF membranes (1 h at 100 Volts) (Immun-Blot PVDF membrane, BIO-RAD). Non specific binding sites were blocked with TBST (Tris-buffered saline and 0.1% Tween-20)

containing 5% skim milk, for 2 h at room temperature. For SLC15A3, electrophoresis and proteins transfer conditions are described in Metzger et al. (2021).

Incubations with primary and secondary antibodies and washing conditions were as described in D'Astous-Pagé et al (2016) for CARNS1, CNDP2 and SLC15A4, and in Metzger et al., (2021) for SLC15A3. Description of primary and secondary antibodies used in the present study, including antibody dilutions used for Western blots, is provided in Supplementary Table 1 (below). Blocking peptides were available for CARNS1 (Immune Biosolutions, YV00230-100) and SLC15A4 (1 µg/µl, Immune Biosolutions, YP00231-100), but not for the CNDP2 and SLC15A3 antibodies. We therefore carried out a preadsorption of the CARNS1 and SLC15A4 primary antibodies with their specific blocking peptides to determine if the observed immunoreactive bands result from specific binding.

Protein detection was performed with the chemiluminescent Amersham™ ECL Select Western Blotting Detection Reagent (Sigma-Aldrich, St. Louis, MO) for CARNS1, CNDP2 and SLC15A4 or with the SuperSignal™ West FEMTO chemiluminescent agent (Thermo Scientific, Schwerte, Germany) for SLC15A3. To evaluate transfer efficiency and equal loading of gels, the PVDF membranes were incubated in Ponceau S (0.2% Ponceau S and 3% trichloroacetic acid, Sigma Aldrich) for CARNS1, CNDP2 and SLC15A4 or in Coomassie Brilliant Blue (Sigma-Aldrich) for SLC15A3 (Metzger et al., 2021). Chemiluminescent signals were detected with the Fusion FX7 fluorescence and chemiluminescence imaging system (Montreal Biotech, Dorval, QC, Canada) for CARNS1, CNDP2 and SLC15A4 or as reported in Metzger et al. (2021) for SLC15A3.

References:

Metzger K, Dannenberger D, Tuchscherer A, Ponsuksili S, Kalbe C (2021) Effects of temperature on proliferation of myoblasts from donor piglets with different thermoregulatory maturities. BMC Mol Cell Biol 22(1):36. https://doi.org/10.1186/s12860-021-00376-4

Tomascova A, Lehotsky J, Kalenska D, Baranovicova E, Kaplan P, Tatarkova (2019) A comparaison of albumin removal procedures for proteomic analyses of blood plasma. Gen Physiol Biophys 38(4):305-314. https://doi.org/10.4149/gpb_2019009

Supplementary Table 1. Primary and secondary antibodies characteristics with optimized conditions used

Proteins[a]	CARNS1	CNDP2	PHT2/SLC15A3	PHT1/SLC15A4
Tissues[b]	Positive: LD muscle<br>Negative: uterus, vagina	Positive: LD muscle<br>Negative: serum, plasma	Positive: LD, RH and ST muscles<br>Negative: plasma, serum	Positive: LD muscle<br>Negative: cerebellum
Primary antibody	Chicken polyclonal to pig CARNS1 (IgY)<br>1 mg/ml, Y00230-001 (Immune Biosolutions)	Rabbit polyclonal to human CNDP2 (IgG), 1 mg/ml, ab204351 (Abcam)	Rabbit polyclonal to mouse SLC15A3 (IgG), 1 mg/ml,<br>Ab113819 (Abcam)	Chicken polyclonal to pig SLC15A4 (IgY)<br>1 mg/ml, Y00231-001 (Immune Biosolutions)
Dilution	1:1000	1:500	1:500	1:1000
Immunogen	aa 379-393<br>RVDRPLRHQSSLPQT UniProt W6CP94_PIG	aa 150 to 250<br>TGQEIPVNVRFCLEG MEESGSEGLDELIFAR KDTFFKDVDYVCISD NYWLGKKKPCITYGL RGICYFFIEVECSNKD LHSGVYGGSVHEAM TDLILLMGSL UniProt Q96KP4 · CNDP2_HUMAN	aa 314-363<br>ILPVMVTLVPYWM YFQMQSTYVLQGL HLHIPNIFRTNPNISL LLRSDSS UniProt Q8BPX9 · S15A3_MOUSE	aa 274-286<br>GEGIGVFQQSSKN UniProt I3LDX0_PIG
Secondary antibody	Alpaca anti-chicken IgY (HRP), 2 mg/ml, AC10008A-DAD (Immune Biosolutions)	Goat anti-Rabbit IgG H&L (HRP), 2 mg/ml, ab205718 (Abcam)	Goat anti-rabbit IgG-HRP, 0.4 mg/ml, sc-2004 (Santa Cruz Biotechnology)	Goat anti-chicken IgY H&L (HRP), 2 mg/ml, ab6877 (Abcam)
Dilution	1:40000	1:10000	1:10000	1:75000
Predicted pig isoforms (kDa)[c]	101.84 (W6CP94)<br>98.90 (A0A5G2QZP0)<br>88.61 (U3MG62)<br>77.98 (A0A480PP83)	52.73 (F1SNL7)<br>48.26 (A0A8W4FIV5)<br>43.72 (A0A4X1U2X2)	62.58 (A0A480MV35)<br>58.43 (F1RIA3)	61.55 (I3LDX0)<br>56.25 (A0A4X1UQG7)
Detection	Chemiluminescent ECL™ Select western blotting detection reagent (Sigma-Aldrich)	Chemiluminescent ECL™ Select western blotting detection reagent (Sigma-Aldrich)	SuperSignal™ West FEMTO chemiluminescent agent (Thermo Scientific)	Chemiluminescent ECL™ Select western blotting detection reagent (Sigma-Aldrich)

[a]CARNS1, carnosine synthase 1; CNDP2, carnosine dipeptidase 2; PHT1, peptide/histidine transporter 2; PHT2, peptide/histidine transporter 2; SLC15A3, solute carrier family 15, member 3; SLC15A4, solute carrier family 15, member 4
[b]LD, *longissimus dorsi*; RH, *rhomboideus*; ST, *semitendinosus*
[c]Predicted pig isoforms (kDa) that can be detected with antibodies used in the present study (UniProt numbers, www.uniprot.org)

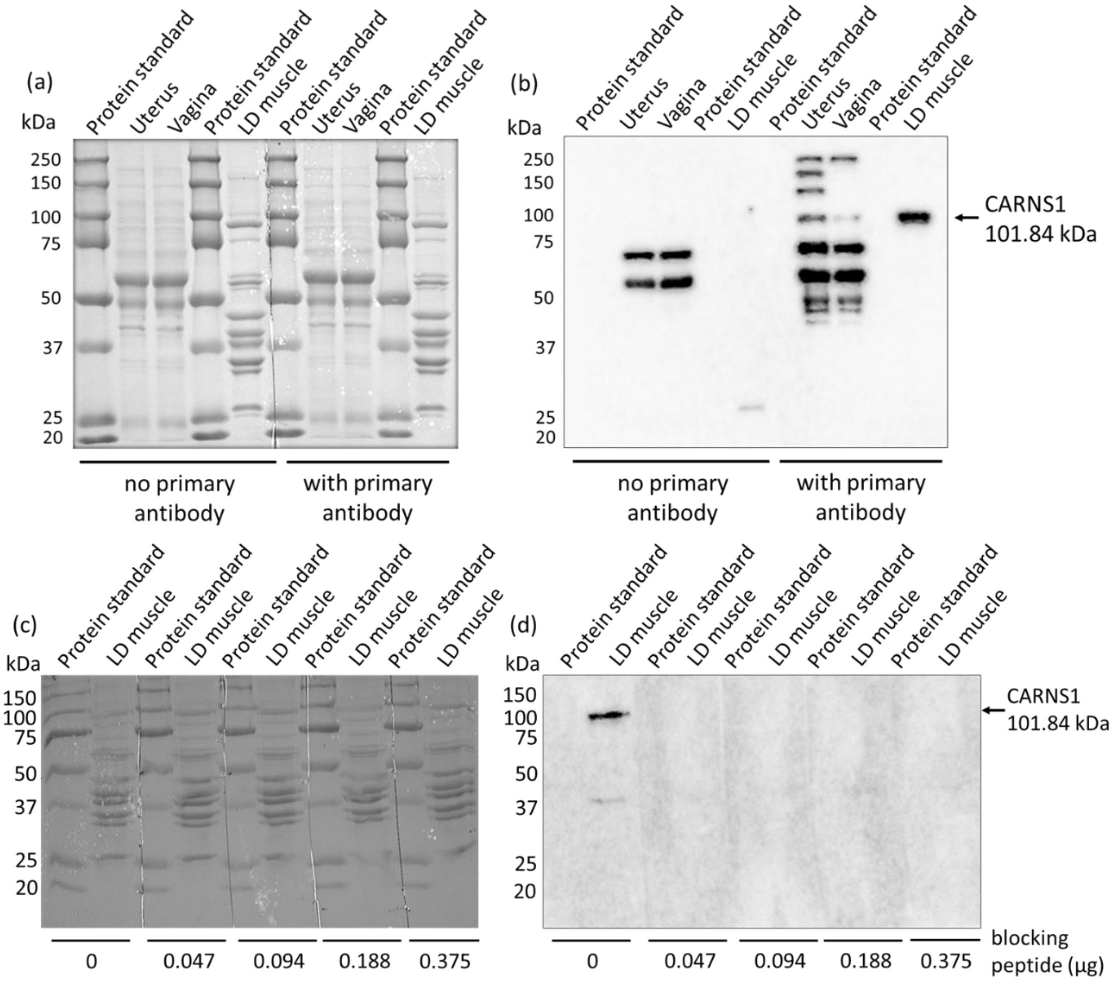

Supplementary Fig. 2 Western blot analysis of the carnosine synthase 1 (CARNS1) protein in pig uterus, vagina and *longissimus dorsi* (LD) muscle. a and b) PVDF membrane stained with Ponceau S and corresponding Western blots; the membrane was cut in two parts to omit the primary antibody (no primary antibody) and to incubate the other part with the primary and secondary antibodies (with primary antibody). c and d) PVDF membrane stained with Ponceau S and corresponding Western blot with the blocking peptide. Membranes were exposed for 2 min. A total of 20 µg (a and b) or 25 µg (c and d) of proteins per well was loaded on gels. Protein standard: Precision Plus Protein All Blue Standards (BIO-RAD). (b) In the LD muscle, a single band was detected at the expected molecular weight for pig CARNS1 (101.84 kDa) and specificity was confirmed when the CARNS1 antibody was incubated in the presence of the blocking peptide (d). In uterus and vagina, several bands are detected including a band at the same molecular weight as that observed for CARNS1 in the LD muscle (b). When compared with the CARNS1 immunoreactive signal found in LD muscle, the corresponding CARNS1 signal intensity is much lower in vagina. The two major bands present in uterus and vagina (between 50 and 75 kDa) are due to non-specific binding of the secondary antibody (b, no primary antibody). Previously reported isoforms for the pig CARNS1 protein that could be detected with the primary antibody are 101.84 kDa, 98.90 kDa, 88.61 kDa and 77.98 kDa (Supplementary Table 1)

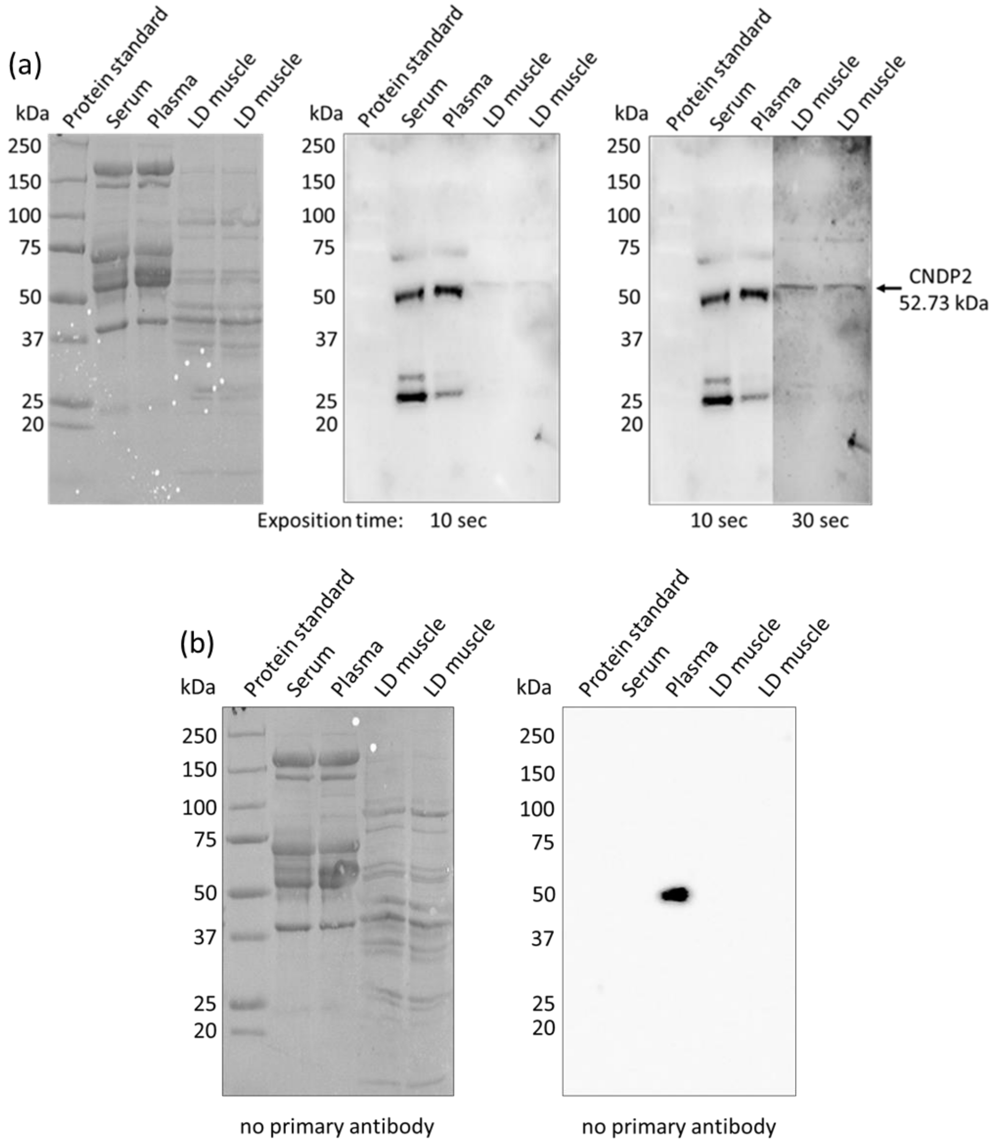

Supplementary Fig. 3 Western blot analysis of the carnosine dipeptidase 2 (CNDP2) protein in pig serum, plasma and *longissimus dorsi* (LD) muscle. (a) Left panel: PVDF membrane stained with Ponceau S; Center: the membrane was exposed for 10 sec; Right panel: the same membrane was exposed for 10 sec (serum and plasma) or 30 sec (LD muscle). A total of 20 µg of proteins per well was loaded on gels. Protein standard: Precision Plus Protein All Blue Standards (BIO-RAD). In the LD muscles, a band was detected at the expected molecular weight for pig CNDP2 (52.73 kDa) and a faint band is also present between 75 and 100 kDa (a). These two bands disappeared when the primary antibody was omitted (b). In serum and plasma samples, stronger signals are detected at ~ 25 kDa and ~ 50 kDa and signals of weaker intensity are present at ~ 28 kDa and ~ 70 kDa (a). With the exception of the 50 kDa band in plasma (non-specific binding of the secondary antibody), all of these bands disappeared when the primary antibody was omitted (b). Previously reported isoforms for the pig CNDP2 protein that could be detected with the primary antibody are 52.73 kDa, 48.26 kDa and 43.72 kDa (Supplementary Table 1)

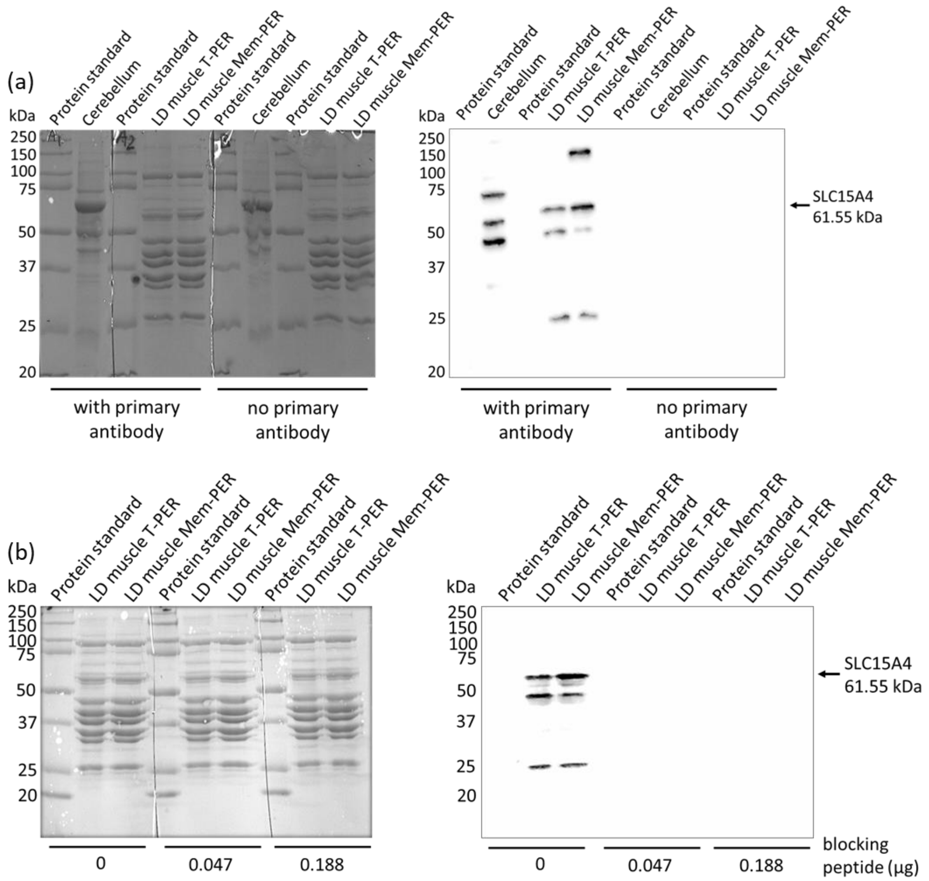

Supplementary Fig. 4 Western blot analysis of the solute carrier family 15, member 4 (SLC15A4) protein in pig cerebellum and *longissimus dorsi* (LD) muscle extracted with T-PER or with Mem-PER Plus. a and b) Left panels: PVDF membranes stained with Ponceau S; Right panels: corresponding Western blots. a) The membrane was cut in three parts: one part to omit the primary antibody (no primary antibody) and the two others to incubate with both the primary and secondary antibodies (with primary antibody). b) The membrane was cut in three parts to incubate with different concentrations of the blocking peptide. Membranes were exposed for 10 sec. A total of 20 µg (a) or 25 µg (b) of proteins per well was loaded on gels. Protein standard: Precision Plus Protein All Blue Standards (BIO-RAD). a and b) In LD muscle T-PER and LD muscle-Mem-PER samples, an immunoreactive band was detected at the expected molecular weight for pig SLC15A4 (61.55 kDa) and its specificity was confirmed when the CARNS1 antibody was incubated in the presence of the blocking peptide. A ~ 150 kDa band is detected in the LD muscle-Mem-PER but not in the LD muscle T-PER sample (a), thus suggesting an effect of the extraction procedure used. Two other bands of lower intensity are also present in LD muscle samples ((a and b) below ~ 50 kDa and ~ 25 kDa) and disappeared when using the blocking peptide (b). In the cerebellum, 3 immunoreactive signals are detected at ~ 48 kDa, ~ 56 kDa and ~ 75 kDa (a). All of these bands disappeared when the primary antibody was omitted (no primary antibody), thus confirming that observed bands are not due to non-specific binding of the secondary antibody. Previously reported isoforms for the pig SLC15A4 protein that could be detected by the primary antibody are 61.55 kDa and 56.25 kDa (Supplementary Table 1)

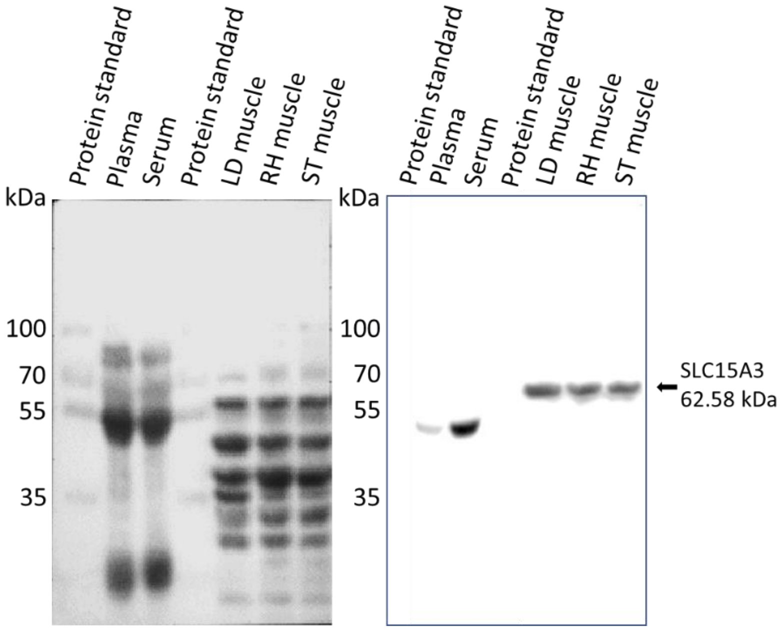

Supplementary Fig. 5 Western blot analysis of the solute carrier family 15, member 3 (SLC15A3) protein in pig plasma, serum and *longissimus dorsi* (LD), *rhomboidus* (RH) and *semitendinosus* (ST) muscles. A total of 30 µg of proteins per well was loaded on gels. Left panel: PVDF membrane stained with Coomassie Brilliant Blue; Membranes were exposed for 5 min. Protein standard: PageRuler™ Plus Prestained Protein Ladder (Thermo Scientific). A single band is detected in skeletal muscle samples (LD, RH and ST) at the expected molecular weight (62.58 kDa) for pig SLC15A3. A single band is also detected in the serum and plasma samples below 55 kDa. Reported isoforms for the pig SLC15A3 protein that could be detected with the primary antibody are 62.58 kDa and 58.43 kDa (Supplementary Table 1)
